# Utilizing metagenomic profiling and machine learning model to identify bacterial biomarkers for major depressive disorder

**DOI:** 10.3389/fpsyt.2025.1539596

**Published:** 2025-02-18

**Authors:** Xuan Wang, Di Cao, Hanlin Zhang, Wei Chen, Jiaxin Sun, Huimin Hu

**Affiliations:** ^1^ Department of Dermatology, Lianyungang Municipal Oriental Hospital, Lianyungang, China; ^2^ Department of Dermatology, The First People’s Hospital of Lianyungang, Lianyungang, China; ^3^ Department of Dermatology, Jieshou City People’s Hospital, Fuyang, China; ^4^ Department of Dermatology, The Affiliated Huai’an Hospital of Xuzhou Medical University and The Second People’s Hospital of Huai’an, Huaian, China

**Keywords:** major depressive disorder, gut bacteria, metagenome, machine learning, diagnosing disease

## Abstract

**Background:**

Major depressive disorder (MDD) is highly heterogeneous, which provides a significant challenge in the management of this disorder. However, the pathogenesis of major depressive disorder is not fully understood. Studies have shown that depression is highly correlated with gut flora. The objective of this study was to explore the potential of microbial biomarkers in the diagnosis of major depressive disorder.

**Methods:**

In this study, we used a metagenomic approach to analyze the composition and differences of gut bacterial communities in 36 patients with major depressive disorder and 36 healthy individuals. We then applied a Support Vector Machine Recursive Feature Elimination (SVM-RFE) machine learning model to find potential microbial markers.

**Results:**

Our results showed that the alpha diversity of the intestinal flora did not differ significantly in major depressive disorder compared to healthy populations. However, the beta diversity was significantly altered. Machine learning identified 8 MDD-specific bacterial biomarkers, with Alistipes, Dysosmobacter, Actinomyces, Ruthenibacterium, and Thomasclavelia being significantly enriched, while Faecalibacterium, Pseudobutyrivibrio, and Roseburia were significantly reduced, demonstrating superior diagnostic accuracy (area under the curve, AUC = 0.919). In addition, the gut bacteria performed satisfactorily in the validation cohort with an AUC of 0.800 (95% CI: 0.6334-0.9143).

**Conclusion:**

This study reveals the complex relationship between gut microbiota and major depressive disorder and provides a scientific basis for the development of a microbiota-based diagnostic tool for depression.

## Introduction

Major depressive disorder (MDD) is a gradually debilitating global mental illness, more than 300 million people of all ages are suffering from depression, the prevalence of depression in China is 4.2%, and it is conservatively estimated that the number of people suffering from depression in China is more than 58 million (1, 2). Major depressive disorder is mainly driven by neuroendocrine, leading to neuroimmunity, metabolism or neurotransmitter imbalance, which is characterized by a low mood, slow thinking, reduced volitional activity, cognitive impairment and somatic symptoms (3, 4). In addition, depression has a serious suicidal tendency. The incidence of suicide among individuals with depression is approximately 10 times higher than in the general population, and approximately one-quarter of patients with depressive disorder have developed suicidal tendencies (5).

The mechanism of depression remains unclear. Studies have shown that the monoamine neurotransmitter hypothesis is the most widely accepted classical hypothesis, which suggests that decreased levels of dopamine (DA), serotonin (5-HT), and other monoamine neurotransmitters are an important mechanism in the onset and development of depression (6, 7). A decrease in gamma-aminobutyric acid (GABA) and an increase in glutamate also play an important role in the pathological development of depression (8, 9). In addition, there is a growing body of literature that supports and characterizes a gut-brain axis and elucidates a potential role for dysfunction of the gut microbiome in major depressive disorder. Animal studies have supported the possibility that dysbiosis (disruption of the microbiome) plays a causative role in depression-like behaviors. Broad-spectrum antibiotic administration in mice leads to dysbiosis, depression-like behaviors, and altered neuronal firing in the hippocampus (10, 11).

Machine learning is a subfield of artificial intelligence that develops models for prediction or decision-making through the analysis of data. The application of machine learning facilitates more effective processing and analysis of microbial datasets. In this study, we employed metagenomic techniques to analyze the intestinal bacterial communities of patients with MDD and healthy individuals and constructed a diagnostic model for MDD based on machine learning algorithms. We hope that machine learning modeling based on the analysis of gut bacterial communities will prove to be a valuable tool in the diagnosis of depression.

## Method

### Data collection

Metagenomic sequencing data from NCBI (SRA accession numbers: PRJNA762199 and PRJNA1083304) were used in this study. There were a total of 36 healthy individuals and 36 patients with MDD in the PRJNA762199 test dataset. There were a total of 20 healthy individuals and 16 patients with MDD in the PRJNA1083304 validation dataset. All patients met the diagnostic criteria for MDD, and the severity of anxiety and depression was assessed using the HAMD-17. And all of these patients were first-onset MDD who had not received medication and had no history of substance abuse.

### Data processing

To avoid the bias caused by different data processing methods, we chose sequence read archive (SRA) for raw sequencing metadata. The raw data in SRA format were converted to FASTQ format using the fastq-dump function of the SRA toolkit. The quality of the sequencing reads was assessed using FASTQC. Tool Kneaddata was first used to perform quality control on the metagenomic sequencing data (Specific parameters: –trimmomatic-options “SLIDINGWINDOW:4:20 MINLEN:50” –bowtie2-options “–very-sensitive –dovetail”). Sequences after quality control were then annotated using Kraken2 based on the bacterial database. Finally, Bracken was used to estimate the abundance of different microbial communities in each sample. All software invocations were performed on a Linux/Ubuntu system using bash commands.

### Data visualization

All downstream data analyses of the metagenome were performed in R software (version 4.3.1). Sample data were normalized using the phyloseq package while filtering out some OTUs with lower abundance. Alpha diversity was analyzed using the vegan package and P < 0.05 was considered statistically significant. Ggplot2 and ggpubr packages were used to visualize alpha diversity. Beta diversity analysis was performed using permute, lattice, vegan, and ape packages, and principal coordinate analysis (PCoA) was plotted based on Bray-Curtis distance. Differences in bacterial communities between the MDD groups and healthy controls were analyzed using Statistical Analysis of Metagenomics Profiles (STAMP) (Welch t-test). Bacterial communities with high specificity and sensitivity were screened using the support vector machine recursive feature elimination (SVM-REF) to discriminate between patients with major depression and healthy individuals. The receiver operating characteristic (ROC) curves were generated with the MedCalc software.

## Results

### Comparison of gut bacteria diversity in patients with MDD and healthy controls

To characterize the gut bacteria of patients with MDD, we analyzed metagenomic sequencing data from different databases from different countries. One of the databases was used for modeling analyses, including 36 MDDs and 36 healthy individuals. Patients with MDD and healthy controls (HCs) were similar in age, sex, and body mass index (BMI) in training cohort (P > 0.05). MDD group had higher total Hamilton Rating Scale for Depression (HAMD-17) scores than the HC group (P < 0.05) (**Table 1**). After analyzing the metagenomic sequencing data, a total of 2,368,263,997 raw reads associated with bacteria were obtained from 72 libraries. An additional database of 16 MDDs and 20 healthy individuals was used for further validation analyses. Patients with MDD and HCs were similar in sex and body mass index (BMI) in validation cohort (P > 0.05). However, there was a difference in age between the two groups. MDD group had higher total Hamilton Rating Scale for Depression (HAMD-17) scores than the HC group in validation cohort (P < 0.05) (**Table 2**).

**Table 1:** Clinical characteristics in MDD and HCs (training cohort).

Variable	MDD (n = 36)	HCs (n = 36)	t/χ2	P
Sex(Male/Female)	19/17	19/17	0.000	1.000^a^
Age (years)	30.83±10.769	33.97±10.476	1.254	0.214^b^
BMI	22.39±4.680	24.63±4.765	1.866	0.067^b^
HAMD-17	21.33±3.610	1.64±1.944	28.822	0.000^b^

a P value for chi-square test.

b P values for two-sample t-test.

**Table 2:** Clinical characteristics in MDD and HCs (validation cohort).

Variable	MDD (n = 16)	HCs (n = 20)	t/χ2	P
Sex(Male/Female)	8/8	7/13	–	0.500^a^
Age (years)	20.19±4.230	23.50±3.348	2.625	0.013^b^
BMI	21.68±3.626	22.17±2.115	0.507	0.615^b^
HAMD-17	26.31±6.620	1.85±2.720	15.054	0.000^b^

a P value for chi-square test.

b P values for two-sample t-test.

**Figure 1:**
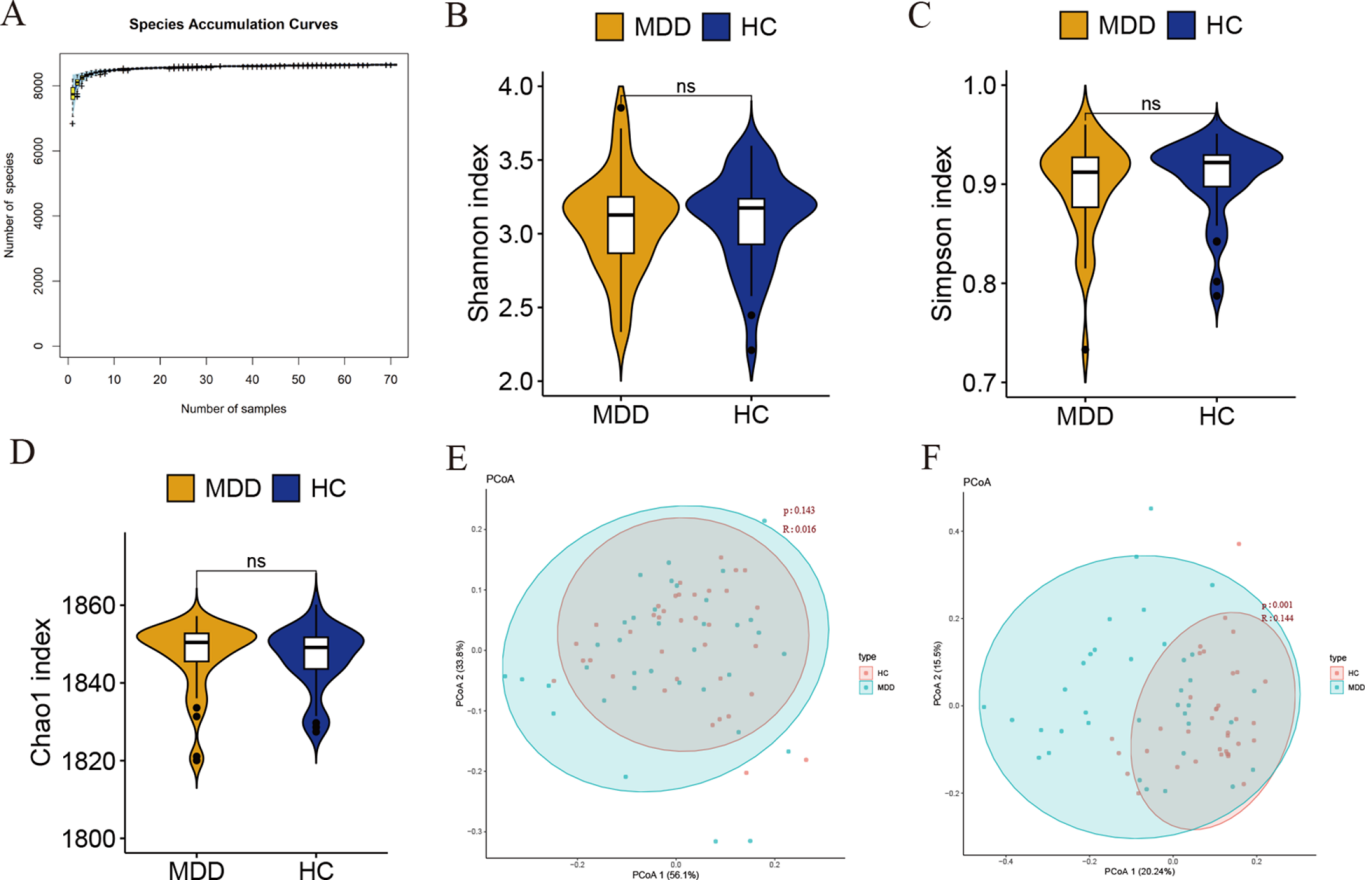
**(A)** Species accumulation curve. The abscissa represents samples and the ordinate represents the number of the cumulative number of species found. **(B–D)** Alpha diversity was estimated by the Shannon index, Simpson, and Chao1 index. Ns indicates not statistically significant difference. **(E, F)** Beta diversity analysis of MDD and healthy controls at the phylum and genus levels.

First, species accumulation curve analysis was performed to verify that the sample size of the experiment was appropriate. The results showed that the species accumulation curve reached a plateau, indicating that the sample size was sufficient to reveal the characteristics of the gut bacteria (**Figure 1A**). We chose the Shannon–Wiener, Simpson, and Chao1 index to calculate the microbial alpha diversity. The results showed that there was no statistically significant difference in the Shannon, Simpson, and Chao1 index between MDD and healthy controls (P > 0.05, Wilcoxon test) (**Figures 1B–D**).

We then calculated the beta diversity between the groups based on the Bray-Curtis distance and performed a principal coordinate analysis (PCoA). Unweighted UniFrac analyses showed that PCoA could discriminate the healthy controls and MDD groups at the genus level (**Figure 1F**). Unfortunately, we did not observe significant differences between the two groups at the phylum level (**Figure 1E**). Taken together, these results suggest that gut bacterial communities may be different between MDD and control groups.

### Composition and differences of gut bacteria in patients with MDD and healthy controls

**Figure 2:**
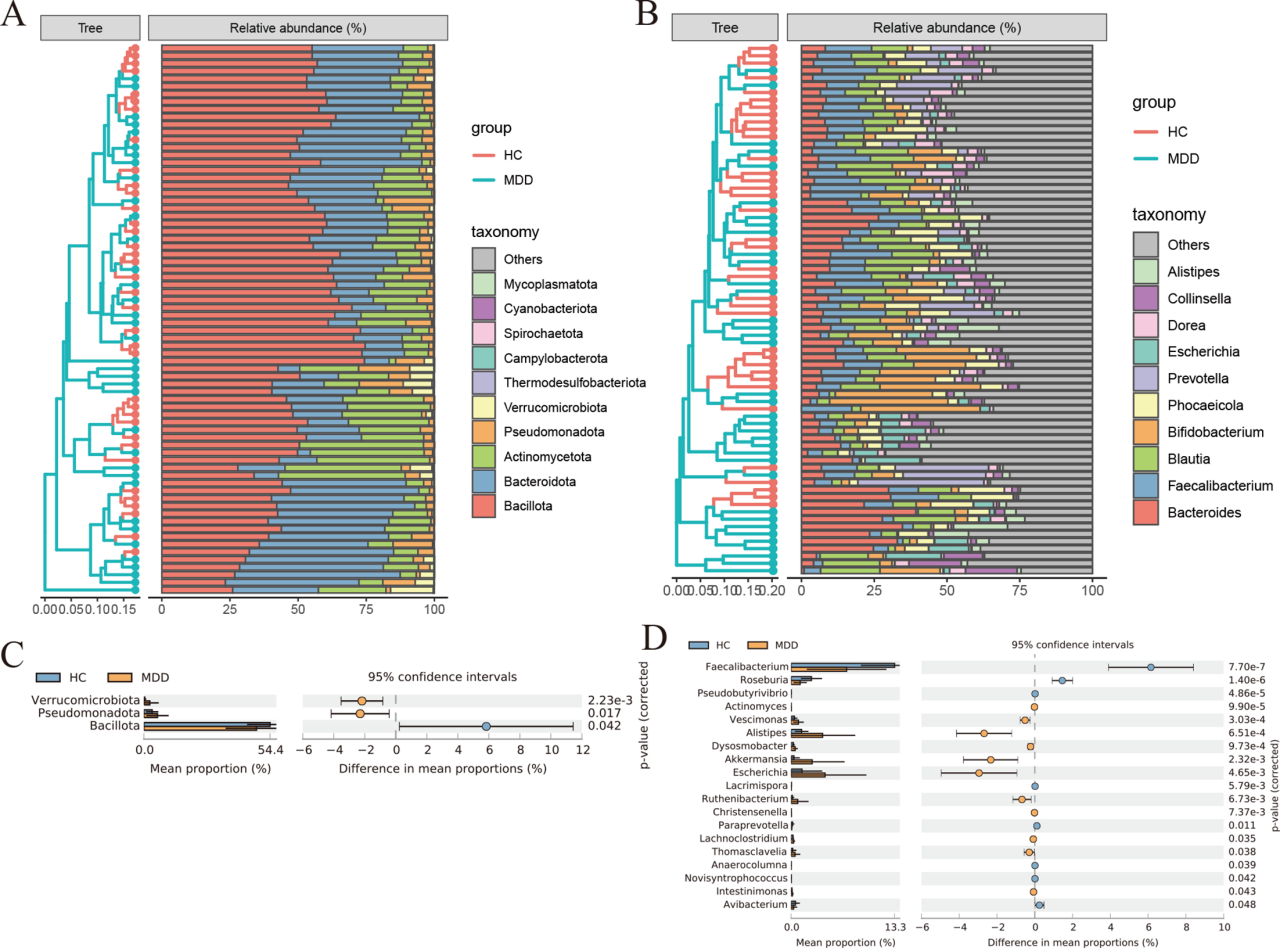
**(A)** Histogram of species abundance at phylum level. **(B)** Histogram of species abundance at genus level. **(C, D)** Analysis of differences in gut bacterial community composition between MDD and healthy controls using STAMP at the phylum and genus levels. Significance values shown were calculated using two-sided Welch t-tests.

At the phylum level, Bacillota, Bacteroidota, and Actinomycetota were the three most abundant bacteria in all groups. At the genus level, Bacteroides, Faecalibacterium, and Blautia were the most abundant bacteria in the gut (**Figures 2A, B**). Statistical Analysis of Metagenomics Profiles (STAMP) was used to identify the gut bacterial communities that distinguish differences between patients with MDD and healthy controls. At the phylum level, Verrucomicrobiota, Pseudomonadota, and Bacillota were significantly different. At the genus level, Faecalibacterium, Roseburia, Pseudobutyrivibrio, Actinomyces, Vescimonas, Alistipes, Dysosmobacter, Akkermansia, Escherichia, Lacrimispora, Ruthenibacterium, Christensenella, Paraprevotella, Lachnoclostridium, Thomasclavelia, Anaerocolumna, Novisyntrophococcus, Intestinimonas, Avibacterium were significantly different. A total of 22 different bacterial communities were identified at the phylum and genus levels. Thirteen bacterial communities were significantly enriched in MDD groups compared to healthy controls, including Verrucomicrobiota, Pseudomonadota, Alistipes, Akkermansia, and Escherichia. Meanwhile, 9 bacterial communities were significantly reduced, including Bacillota, Faecalibacterium, Roseburia, Paraprevotella, Avibacterium, etc. (**Figures 2C, D**).

### Screening for bacterial biomarkers to differentiate MDD from healthy controls

**Figure 3:**
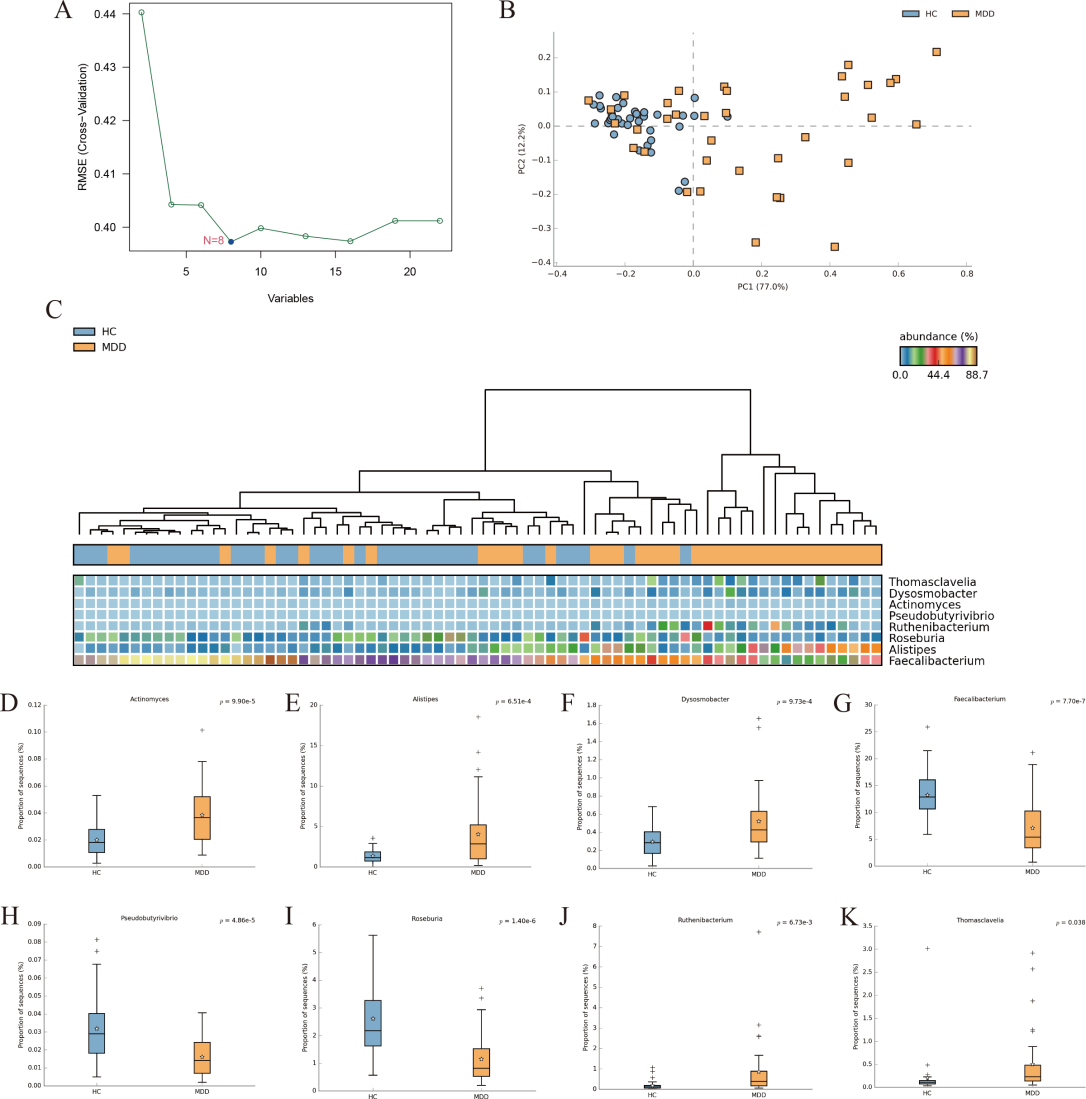
**(A)** SVM-REF model for screening bacterial biomarkers that distinguish MDD from healthy individuals. **(B)** PCoA of the 8 differential bacterial communities. **(C)** Heat map of the 8 differential bacterial communities. **(D–K)** Differential analysis of the abundance of characteristic gut bacterial communities was performed using STAMP (Welcht’s test).

The SVM-REF algorithm was used to screen potential microbial markers that could be used as diagnostic indicators from different bacterial communities of MDD. For the SVM-REF algorithm, the classifier error is minimized when the number of features is 8. Eight characteristic bacterial communities were finally identified (**Figure 3A**). The PCOA results showed that characteristic bacterial communities could distinguish healthy individuals with MDD (**Figure 3B**). In the heatmap we can observe the difference in the abundance of the 8 bacterial communities between MDD and the healthy groups (**Figure 3C**). Then, we compared the abundance of the characterized bacterial communities and showed that the abundance of the 8 bacterial communities was significantly different between MDD and healthy individuals (**Figures 3D–K**).

**Figure 4:**
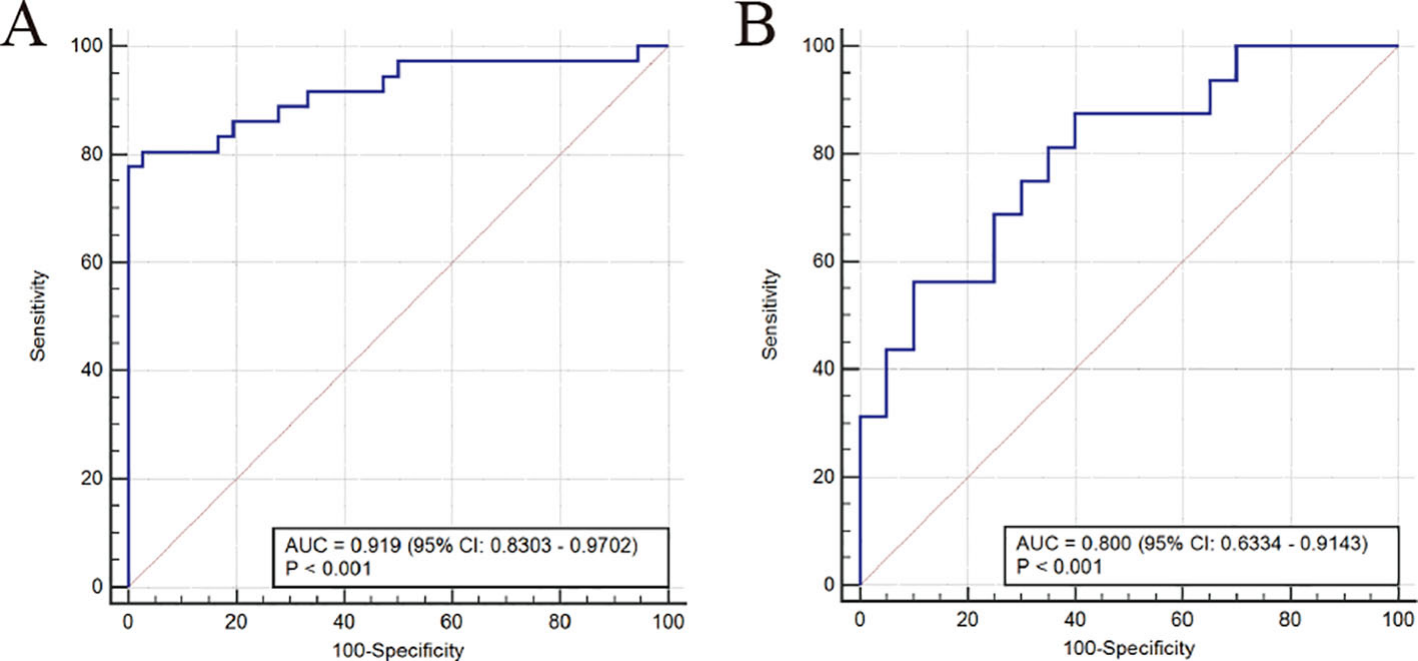
Area under the ROC curve (AUC) was used to evaluate the diagnostic performance. ROC curve of the models in the training group **(A)** and validation group **(B)**.

Finally, we evaluated the predictive performance of the bacterial communities for the diagnosis of MDD using ROC curves. The results showed that the model constructed by the SVM-REF algorithm had a good predictive performance with an AUC value of 0.919 (95% CI: 0.8303-0.9702), indicating that these characteristic bacterial communities can be used as diagnostic indicators for MDD (**Figure 4A**). To further confirm the diagnostic potential of the bacterial communities in other samples, an independent test was conducted using an external validation cohort from Shanxi Province to confirm the reliability of the model. To further confirm the diagnostic potential of the gut bacteria, we validated the reliability of the model using an external validation cohort from Shanxi Province. The results showed that the validation cohort had an AUC of 0.800 (95% CI: 0.6334-0.9143), which was a good prediction (**Figure 4B**). Therefore, we can conclude that the characteristic bacterial communities have good predictive performance for the diagnosis of MDD.

## Discussion

Depression, as one of the most prevalent psychiatric disorders globally, exhibits a sharp annual increase in incidence rates and is the leading cause of global disability burden (12, 13). Clinical manifestations of major depressive disorder (MDD) are diverse and complex, with core characteristics including mood dysregulation, cognitive decline (such as memory impairment), motor function impairment (presented as reduced physical capacity), diminished energy (with increased fatigue), and a lowered sense of self-worth. Moreover, patients with MDD face a high risk of severe disability, which further exacerbates the negative impact on individual quality of life and socio-economic outcomes (14).

The gut microbiota plays a pivotal role in maintaining physiological functions in the gastrointestinal tract, including but not limited to regulating intestinal secretion, facilitating digestive processes, enhancing nutrient absorption, and synthesizing various vitamins and short-chain fatty acids such as acetate, butyrate, propionate, and lactate (15). Furthermore, the gut microbiota is involved in the biosynthesis of neurotransmitters and their precursors, including dopamine, norepinephrine, γ-aminobutyric acid (GABA), and acetylcholine. They also secrete and upregulate a range of essential proteins and metabolites that play key roles in the release of neuropeptides and gut hormones, thereby impacting neuroendocrine signaling. Additionally, the gut microbiota, through its metabolic activities and immune-modulatory functions, can finely tune the host’s immune responses, including the promotion of anti-inflammatory and immune tolerance mechanisms, as well as the regulation of inflammatory signaling pathways. These functions are crucial for maintaining the homeostasis of the gut-brain axis and overall health (16). Numerous previous studies have clearly indicated that the imbalance of the structure and function of the gut microbiota, known as dysbiosis, along with the associated dysfunction of the microbiota-gut-brain axis, may be a direct pathological mechanism in the occurrence and development of depression. These studies reveal the complex interplay between the gut microbiota and the central nervous system, as well as their key roles in mood regulation and behavioral expression (15–19).

In this study, we employed Statistical Analysis of Metagenomic Profiles (STAMP) to identify and differentiate key bacterial populations within the gut microbiota between patients with major depressive disorder (MDD) and healthy individuals. Additionally, we utilized a Support Vector Machine Recursive Feature Elimination (SVM-REF) model, which, while retaining the advantages of the Support Vector Machine (SVM), optimizes variable selection in the predictive model by reducing the number of feature vectors (20). Leveraging the superior predictive capacity of the SVM-REF model, we identified eight bacterial communities with statistically significant differences at the phylum and genus levels, which are considered potential microbial biomarkers for the diagnosis of MDD. Specifically, the relative abundance of Alistipes, Dysosmobacter, Actinomyces, Ruthenibacterium, and Thomasclavelia was significantly higher in MDD patients compared to healthy controls, while the abundance of Faecalibacterium, Pseudobutyrivibrio, and Roseburia was significantly reduced in MDD patients.

The relative abundance of five bacterial communities is elevated in patients with major depressive disorder, as detailed below: Alistipes, an indole-positive organism, can reduce the bioavailability of serotonin and metabolizes glutamate to γ-aminobutyric acid (GABA) through the expression of glutamate decarboxylase, and an increase in its abundance may disrupt the function of the gut-brain axis (21). Although literature on Dysosmobacter is scarce, studies suggest that it may modulate immune responses through interactions with the host’s immune system (22). Actinomyces has been reported to be more abundant in patients with major depressive disorder (23). Bacteria of the genus Ruthenibacterium are implicated in COVID-19 pathogenesis, with affected patients exhibiting reduced immune cell levels and refractory hypoxemia (24). Thomasclavelia may be associated with chronic inflammatory diseases of the gut (25).

The abundance of the following three bacterial genera is significantly lower in patients with major depressive disorder: bacteria of the genus Faecalibacterium are associated with anti-inflammatory activity, particularly in inflammatory bowel disease, where a reduction in their numbers correlates with inflammatory conditions. Furthermore, Faecalibacterium species can produce short-chain fatty acids such as acetate, propionate, and butyrate. This genus can also modulate the host’s immune system, including upregulating the expression of IL-10 and enhancing T-cell proliferation, playing a significant role in the immune regulation of the gut-brain axis (26). In non-psychiatric conditions such as spinal cord injury and drug-induced liver injury, the metabolic pathways of gut bacteria are impaired, and the abundance of Pseudobutyrivibrio is consistently lower (27, 28). Roseburia, capable of producing short-chain fatty acids, significantly increases the levels of 5-hydroxytryptamine (5-HT) in the brain and colon, inhibits the expression of rate-limiting enzymes, and can reverse the stress-induced conversion of tryptophan to kynurenine in the brain and colon, demonstrating antidepressant functions. Additionally, Roseburia has been confirmed to efficiently predict major depressive disorder in adolescents (29).

When assessing the risk of developing major depressive disorder, the predictive power of individual bacterial populations is limited and does not provide accurate and reliable results. In contrast, the eight bacterial populations selected in this study, due to their significant differences in composition, exhibit higher specificity and sensitivity, thereby being more effective in predicting major depressive disorder. Furthermore, through external validation analyses, the predictive model constructed using these eight bacterial populations was able to accurately identify patients with major depressive disorder, confirming the model’s high predictive accuracy and practical utility.

This study successfully identified eight bacterial populations significantly associated with major depressive disorder at the phylum and genus levels using Statistical Analysis of Metagenomic Profiles (STAMP) and Support Vector Machine Recursive Feature Elimination (SVM-REF) models. These bacterial populations, as potential microbial biomarkers, demonstrate significant application value in the diagnosis of major depressive disorder. The study not only unravels the complex relationship between the gut microbiota and major depressive disorder but also provides a scientific basis for the development of microbiome-based diagnostic tools for depression, highlighting the importance of multi-population analysis in enhancing predictive accuracy and practicality. However, this study also has certain limitations, such as: limited sample size and cross-sectional design cannot determine causality. Future studies should focus on addressing these limitations, by increasing the sample size, adopting a longitudinal study design, and considering more potential confounding factors, to further verify these findings and explore their potential for application in clinical practice.

## Data Availability

The datasets presented in this study can be found in online repositories. The names of the repository/repositories and accession number(s) can be found in the article/supplementary material.
